# Editorial Note: Development and validation of a quantitative Proximity Extension Assay instrument with 21 proteins associated with cardiovascular risk (CVD-21)

**DOI:** 10.1371/journal.pone.0358531

**Published:** 2026-09-18

**Authors:** The PLOS One Editors

The *PLOS One* Editors issue this Editorial Note to clarify statements in the article [1] to address their potential interpretation as implicitly suggesting equivalence between the new reported assay and gold standard immunoassays. Such an interpretation is not supported by the analyses and results reported in this article. A member of the *PLOS One* Editorial Board reviewed the analyses and noted that while the reported inter-assay correlation analyses and Bland-Altman analysis appear technically appropriate, the results suggest a relatively large variability between measurements, such that together they provide support for preservation of patient ranking/prognostic stratification but limited evidence of close quantitative agreement between assays.

The last author provided the following clarifications:

The CVD-21 chip examined in this article was evaluated as an initial multiplex research tool for cardiovascular risk stratification and future decision support, not as an established clinical diagnostic test or as a validated replacement for conventional single-analyte immunoassays. The article presents analytical characterization and limited method comparisons with conventional immunoassays for selected biomarkers, primarily for calibration and contextual interpretation. However, these analyses do not constitute a formal agreement, equivalence, or interchangeability study between CVD-21 and conventional immunoassays. The main clinical comparison concerns prognostic performance and risk discrimination. This interpretation is also supported by the fact that one key marker, troponin, was measured by a conventional high-sensitivity immunoassay in the prognostic models. Thus, the clinical findings should primarily be interpreted as prognostic model comparisons, rather than as evidence that CVD-21 assays can analytically replace conventional immunoassays. The authors acknowledge that the phrase “may provide an alternative to established single biomarker assays” could be misinterpreted; however, it refers to the potential of multiplex PEA-based prognostic profiling rather than to established analytical interchangeability with conventional assays.

Note: During editorial follow-up the last author notified PLOS that the corresponding author is deceased. Correspondence regarding this article may be sent to the last author, lars.wallentin@ucr.uu.se.
